# Essential oil constituents of *Illicium griffithii* and its antimicrobial activity

**DOI:** 10.4103/0973-1296.66938

**Published:** 2010

**Authors:** A. Saraswathy, R. Shakila, S. Mercy Lavanya, A. Arunmozhidevi

**Affiliations:** Department of Chemistry, Arignar Anna Hospital Campus, Arumbakkam, Chennai-600 106, Tamil Nadu, India; 1 Microbiology, Captain Srinivasa Murti Research Institute for Ayurveda & Siddha Drug Development (CCRAS), Arignar Anna Hospital Campus, Arumbakkam, Chennai-600 106, Tamil Nadu, India

**Keywords:** Antifungal activity, essential oil, gas chromatography-mass spectroscopy, *Illicium griffithii*, linalool

## Abstract

The essential oil of the fruit of *Illicium griffithii* Hook f. et Thoms. was extracted using Clevenger’s apparatus. Forty-one compounds were characterized by gas chromatography-mass spectroscopy (GC-MS). 4-Methyl-6-(2-propenyl)-1,3-benzodioxole was characterized as the major constituent, followed by linalool amongst the volatile constituents. The essential oil was found to be effective against *Aspergillus niger, Penicillium* spp. and *Saccharomyces cerevisiae* and possessed considerable activity against *Staphylococcus aureus* and was inactive against *Klebsiella pnemoniae, Pseudomonas aureginosae, Proteus vulgaris* and *Escherichia coli*.

## INTRODUCTION

*Illicium griffithii* Hook. f. et Thoms. belongs to Illiciaceae family. It is a large shrub o13f 3–4.5 m height, found at an altitude of 1400–1700 m in northeastern states of India, Khasi hills and Bhutan. Its fruit is composed of compressed, beaked, incurved carpels, each containing one seed arranged in a single whorl. The fruit has a slightly aromatic, bitter and astringent taste. It is used as a stimulant and carminative.[1] Linalool, limonene, α-pinene, 1,8-cineole, β-methoxyphenyl acetone, terpinen-4-ol, (E)-anethole, safrole, germacrene B, cadinol, myristicin, α-selinene, δ-selinene, α-santalene, β-phellandandrene,[2–4] elemicin, (E)-caryophyllene and eugenol derivatives[5] were reported previously as the chemical constituents of the essential oil of *I*. *griffithii*, among which linalool was the major constituent.[2] *p*-Menth-1(7),4(8)-diene-3-O-β-D-glucoside was also reported to be present in the fruit.[6] The present study reports 41 volatile constituents from the essential oil of the fruit of *I*. *griffithii* and its antimicrobial activity.

## MATERIALS AND METHODS

### Plant material

The dried fruit of I. griffithii was procured from Arunachal Pradesh, India, and was identified by National Botanical Research Institute, Lucknow. A voucher specimen (no. I/145ICMR88) was deposited in the museum of this institute.

### Instrument

Shimadzu GC 2010 was the instrument used for gas chromatography-mass spectroscopy (GC-MS) analysis. The constituents were identified by comparison of the mass fragments with the spectrum library NIST/EPA/NIH.

### Test organisms

Organisms such as *Klebsiella pnemoniae* (ATCC 700603), *Pseudomonas aureginosae* (ATCC 27853), *Escherichia coli* (ATCC 25922), *Proteus vulgaris* (ATCC 9484) and *Staphylococcus aureus* (ATCC 25923) were used for the study. The organisms were procured from Christian Medical College, Vellore, and were maintained by serial sub-culturing every month on nutrient agar slants and incubating at 37°C for 18–24 hours. The cultures were stored under refrigerated condition. The antifungal activity of the oil was tested against *Aspergillus niger, Penicillium* spp. (both were isolated from soil) and *Saccharomyces cerevisiae* (isolated from dough).[7–9] Isolation and identification of the fungus was done by the microbiologist of this institute.

### Extraction of essential oil

Exactly 200 g of the dried fruit of *I*. *griffithii* was coarsely powdered and transferred in to a 2-1 round bottom flask. Sufficient amount of water was added and fixed with Clevenger’s apparatus. This was boiled for 4 h and the steam-distilled essential oil (yield 0.85%) was collected, then dried over anhydrous sodium sulfate, transferred into an airtight sample tube and stored at 8°C.

### Gas chromatography-mass spectroscopy analysis of the essential oil

**Figure 1:**
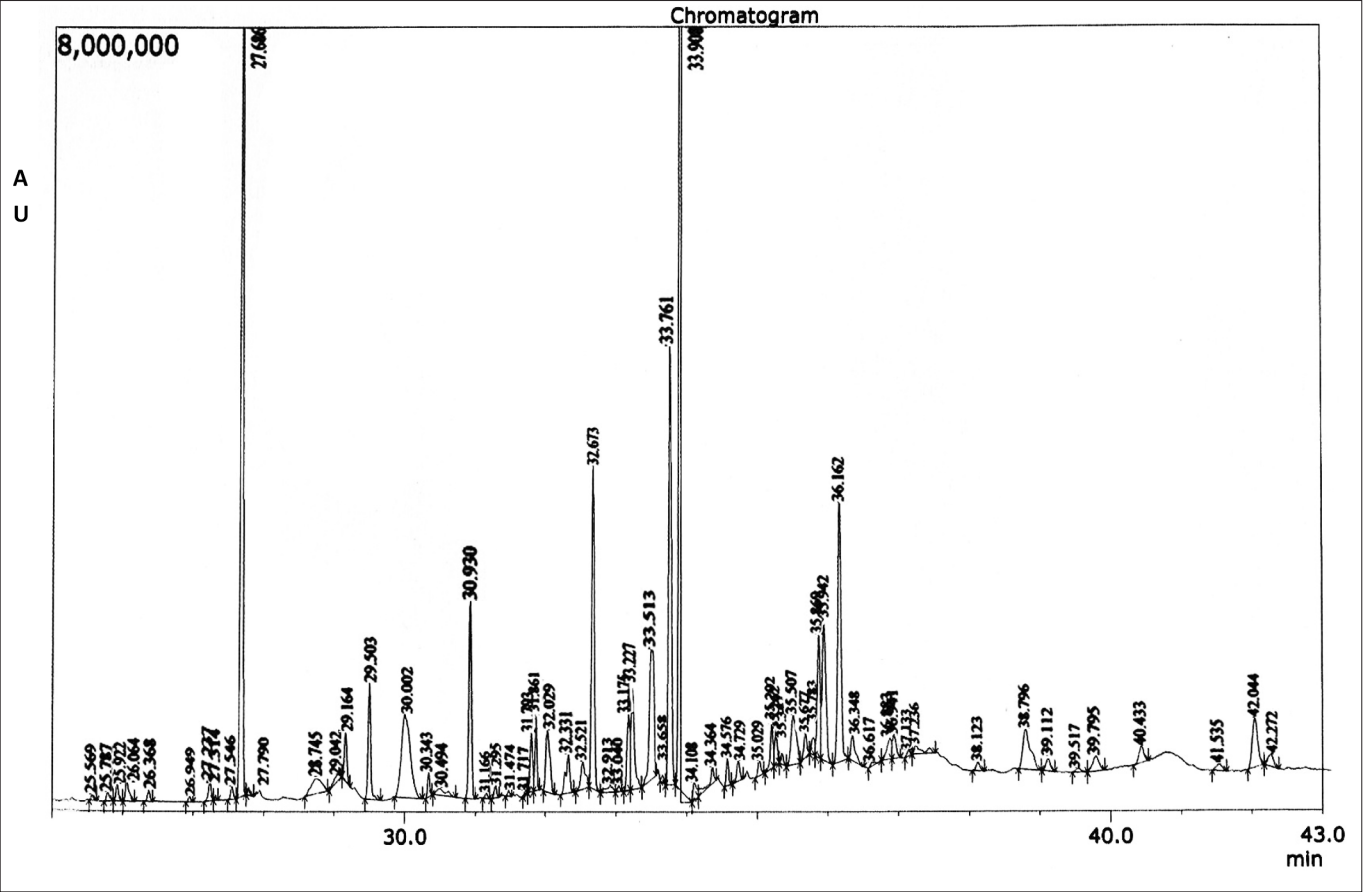
GC-MS chromatogram of essential oil of *I*. *griffithii fruit*

One microliter of the essential oil of the fruit was injected into GC. The injector temperature was maintained at 250°C. The detector used was flame ionization detector which was maintained at 280°C. The pressure of the carrier gas, nitrogen, was kept at 10 psi. The oven temperature was set at 60–280°C with a gradual increment of 10°C/min. The injected oil was eluted in the DB-5 MS column of 30 m length and 0.25 mm inner diameter and the eluted constituents were detected by flame ionization detector and the GC chromatogram was recorded [Figure 1].

### Antibacterial activity

Antibacterial activity was determined by the well diffusion method.[10] Petri plates containing 25 ml of nutrient agar medium were seeded with a 24-h culture of the bacterial strains. The inoculum’s size was adjusted so as to deliver a final inoculum of approximately 108 colony-forming units (CFU/ml). Wells (6 mm diameter) were made on solidified inoculated nutrient agar plates by using sterile plunger. Ten and 20 µl of the oil were transferred aseptically to the subsequent wells and labeled. Standard disc of ampicillin 30 µg (positive control) was placed on the inoculated plate to evaluate the potency of the oil. The plates were left undisturbed for 15 min at room temperature to aid in seeping of the oil and then the plates were incubated at 37°C for 24 h. The zone of inhibition was measured in millimeters.

### Antifungal activity

The antifungal activity was tested by agar diffusion method.[11–13] Petri plates containing 25 ml of Sabouraud dextrose agar (SDA) medium were seeded with a 7-day old culture of fungus organisms. Wells (6 mm diameter) were made on solidified inoculated SDA plates by using sterile plunger. Ten and 20 µl of the oil were transferred aseptically to the subsequent wells and labeled. Standard disc of 30 µg of amphotericin B was loaded as a reference antifungal drug. The plates were incubated at 25 ± 2°C for 5 days. The zone of inhibition was measured in millimeters.

## RESULTS

The volatile oil constituents along with their retention time and percentage obtained from the GC-MS analyzer are given in Table 1. The spectrum obtained is shown in the Figure 1. Forty-one constituents were identified by the detector.

**Table 1:** GC-MS data of essential oil of *I*. *griffithii fruit*

Compounds	Retention time (min.)	Area %
α-Terpinene	25.569	0.07
β-Terpinene	25.787	0.14
δ-Terpinene	25.922	0.23
Eucalyptol	26.064	0.62
α-Terpinolene	26.949	0.07
Linalool oxide	27.237	0.28
Linalool	27.686	12.05
δ-Candinene	28.745	0.81
α-Candinol	29.042	3.86
4-Terpineol	29.164	0.38
Nerol	30.343	0.31
β-Eudesmol	30.494	0.31
Safrol	30.930	2.47
Phellandral	31.166	0.07
α-Cubene	31.295	0.15
Thymol	31.474	0.07
α-Ylangen	31.717	0.04
Copene	31.793	0.76
Nerol acetate	31.861	0.86
β-Bourbonen	32.028	1.23
α-Gurjunene	32.331	0.83
Calarene	32.521	0.70
Caryophyllene	32.673	4.43
Valencene	32.913	0.24
Cadinene	33.040	0.08
α-Caryophyllene	33.175	1.18
Isoledene	33.227	1.73
δ-Murolene	33.513	3.18
4-Methyl-6-(2-propenyl)-1,3-benzodioxole	33.908	22.64
2-Methyl eicosane	34.108	0.35
α-Calacorene	34.364	0.42
Elemol	34.729	0.33
Ledol	35.029	0.25
Spathulenol	35.262	0.43
(–)-Globulol	35.347	0.14
Di-epi-α-cedrene	35.677	0.40
Fonenol	35.783	0.31
Epizonaren	35.869	1.86
Eugenol	36.941	0.44
Rimuene	39.795	0.51
Suberosin	42.272	0.34

The essential oil obtained from *I*. *griffithii* was tested for its antibacterial activity against five strains, viz., *K*. *pnemoniae* (ATCC 700603), *Ps*. *aureginosae* (ATCC 27853), *E*. *coli* (ATCC 25922), *Pr*. *vulgaris* (ATCC 9484) and *St*. *aureus* (ATCC 25923) and for the antifungal activity against *A*. *niger*, *Penicillium* spp. and *Sa*. *cerevisiae*. Tables 2 and 3 show the results of the observed antibacterial and antifungal activities, respectively.

**Table 2:** GC-MS data of essential oil of *I*. *griffithii fruit*

Organisms	Inhibition zone (mm)		
	Ampicillin (30 µg)	Essential oil (10 µl)	Essential oil (20 µl)
*K. pneumoniae*	14	—	—
*Ps. aeruginosae*	13	—	—
*E. coli*	13	—	—
*Pr. vulgaris*	14	—	—
*St. aureus*	14	14	14

**Table 3:** Antifungal activity of essential oil of *I*. *griffithii fruit*

Organisms	Inhibition zone (mm)		
	Ampicillin (30 µg)	Essential oil (10 µl)	Essential oil (20 µl)
*A. niger*	12	13	13
*Penicillium spp.*	10	9	9
*Sa. cerevisiae*	11	10	10

## DISCUSSION

Of the 41 constituents identified, the percentage content of 4-methyl-6-(2-propenyl)-1,3-benzodioxole and linalool were observed to be 22.64 and 12.05%, respectively, whereas the content of caryophyllene, safrol, β-bourbonen, isoledene, δ-murolene, α-candinol, epizonaren were found to be <5%. Other constituents were <1%.

Ten and 20 µl of the oil showed antibacterial activity only against *St*. *aureus* and the inhibition zone was 14 mm and the positive control showed the inhibition zone ranging from 13 to 14 mm against *K*.*pneumoniae*, *Ps*. *aureginosae*, *E*. *coli*, *Pr*. *vulgaris* and *St*. *aureus*. There was no considerable change in inhibition found by increasing the concentration of the oil and thus the bacterial strains like *K*. *pneumoniae*, *Ps*. *aureginosae*, *E*. *coli*, *Pr*. *vulgaris* were susceptible to the oil.

Ten and 20 µl oil showed appreciable antifungal activity against *A*. *niger*, *Penicillium* spp. and *Sa*. *cerevisiae*. The inhibition zone of the oil for both the concentrations varied from 9 to 13 mm while for the positive control it was in the range of 10–12 mm. The results of the antifungal activity are shown in Table 3.

## CONCLUSION

The essential oil isolated from *I*. *griffithii* was found to contain 41 volatile constituents, of which 4-methyl-6-(2-propenyl)-1,3-benzodioxole and linalool were the major compounds. The oil has significant activity against spoilage fungus *A*. *niger*, *Penicillium* spp. and *Sa*. *cerevisiae* and was active against the bacterial strain *St*. *aureus*. Therefore, it can be concluded that the oil can effectively be used as an antifungal agent and a food preservative, after detailed research.
